# Towards Generating Authentic Human-Removed Pictures in Crowded Places Using a Few-Second Video

**DOI:** 10.3390/s24113486

**Published:** 2024-05-28

**Authors:** Juhwan Lee, Euihyeok Lee, Seungwoo Kang

**Affiliations:** 1 Lululab Inc., Seoul 06054, Republic of Korea; jh.lee@lulu-lab.com; 2 Department of Computer Science and Engineering, Graduate School, Korea University of Technology and Education, Cheonan 31253, Republic of Korea; euihyeok.lee@misl.koreatech.ac.kr; 3 School of Computer Science and Engineering, Korea University of Technology and Education, Cheonan 31253, Republic of Korea

**Keywords:** human removal, specific object removal, image segmentation, image inpainting

## Abstract

If we visit famous and iconic landmarks, we may want to take a photo of them. However, such sites are usually crowded, and taking photos with only landmarks without people could be challenging. This paper aims to automatically remove people in a picture and produce a natural image of the landmark alone. To this end, it presents Thanos, a system to generate authentic human-removed images in crowded places. It is designed to produce high-quality images with reasonable computation cost using short video clips of a few seconds. For this purpose, a multi-frame-based recovery region minimization method is proposed. The key idea is to aggregate information partially available from multiple image frames to minimize the area to be restored. The evaluation result presents that the proposed method outperforms alternatives; it shows lower Fréchet Inception Distance (FID) scores with comparable processing latency. It is also shown that the images by Thanos achieve a lower FID score than those of existing applications; Thanos’s score is 242.8, while those by Retouch-photos and Samsung object eraser are 249.4 and 271.2, respectively.

## 1. Introduction

Imagine that you are at the Leaning Tower in Piazza del Duomo, Pisa, one of the famous landmarks everyone wants to visit. The desire to preserve memories or showcase the landmark to acquaintances motivates the quest for the perfect shot. However, such famous sites are usually crowded, making it challenging to capture an ideal image of the landmark without people in the background. What if a camera application on your smartphone could automatically remove people from the scene and produce a wonderful picture of the landmark alone?

One of the essential requirements for such an application is that the generated human-removed image should be of high quality so that users perceive it as a natural scene. Existing smartphone applications, such as Retouch-photos [1], Spectre [2], and Samsung object eraser [3], while potentially viable, require the users’ manual processing, which could burden them. In addition, our preliminary study [4] uncovered that the quality of certain parts of the resulting image was degraded significantly. This may be attributed to the complexity of certain regions in which people should be removed or the movement of people to obtain the desired images. It could lead to a poor user experience.

In this paper, a system to generate authentic human-removed images in crowded places, namely Thanos, is proposed. The system is built to run on mobile devices, such as smartphones, for easy access and interaction with users while utilizing external server resources to perform computationally intensive processing. Furthermore, Thanos can produce human-removed images with only a negligible user burden, i.e., capturing a 5-s video without requiring manual specification of the human area. It maximizes the utilization of available data within the video to increase the quality of the resulting images.

The proposed system is developed on top of existing works for image segmentation and inpainting, but it should address several issues. First, while deep learning-based image segmentation techniques can be employed to identify human-related areas automatically in a given image, image segmentation is imperfect [5,6]; the methods often do not accurately identify all human regions. Several peripheral parts of people, such as bags and shoes, hands and feet, often remain after human segmentation. Second, a deep learning-based image inpainting technique is employed to restore cropped areas identified as human. However, the image inpainting technique often results in poor-quality restoration of the part around complex background patterns, large holes, and image boundaries [7,8]. Third, deep learning-based image inpainting techniques usually require high computational costs [8,9].

We develop a multi-frame-based recovery region minimization method to address the issues above. It expands and crops the regions of the identified humans through image segmentation on multiple frames, which can be obtained from a few seconds of video. Information from these frames is aggregated to obtain actual scenes and achieve more natural results. However, in some cases, such as when there is minimal movement of people within a video, cropped regions identified in the initial step may remain. In this case, these regions are designated as Regions of Interest (ROIs), and image inpainting is performed only for these areas rather than processing the entire image.

It is shown that Thanos outperforms alternative methods regarding image quality with comparable processing latency. Furthermore, compared to the alternatives, the lowest FID [10] scores are achieved by the proposed method. It is also shown that Thanos generates more natural human-removed images regarding FID scores compared to two existing applications, Retouch-Photos and Samsung object eraser. The FID scores for Thanos, Retouch-Photos, and Samsung object eraser are 242.8, 249.4, and 271.2, respectively.

The contributions of this paper can be summarized as follows. First, we present Thanos, a system that produces natural-looking human-removed images. We carefully design our system to enable users to achieve their desired results with minimal effort using their smartphones. Second, we propose a novel pipeline for removing humans within a short video based on information aggregation from multiple frames. This approach allows for more natural-looking images while also achieving efficiency in terms of computational resources. Third, we present Thanos’s performance in terms of image quality and processing latency. Furthermore, we show that Thanos outperforms an existing application, Retouch-Photos.

The rest of this paper is organized as follows: The related work is discussed in Section 2. The Thanos system architecture is presented in Section 3. The design and implementation of the main system components are detailed in Section 4. In Section 5, our performance evaluation is shown. Finally, the paper is concluded in Section 7.

## 2. Related Work

In this section, previous works with similar objectives to our work are discussed. Existing methods that could be adopted for the key component of our proposed system are also discussed.

### 2.1. Object Removal

Several services/applications have been developed to remove specific objects from images, such as Retouch-photos [1] and Samsung object eraser [3]. Shetty et al. [11] and Dhamo et al. [12] have also proposed methods for generating specific target-removed images. However, these works often require users to manually specify the target object, which can be time-consuming and impractical. Moreover, when the background behind the object is complex, the results may not appear natural. The Spectre [2] provides a feature to eliminate moving objects by analyzing the frames captured over a few seconds. However, the output quality may not be optimal when the camera is unstable and removing stationary objects is impossible.

Din et al. [13], HiMFR [14], and Sola et al. [15] conducted a study on removing face masks from the images of people wearing masks. They adopted object detection and image completion steps. However, their approach only targets a relatively simple and similar pattern as a person’s head wearing a face mask. In contrast, our target images typically involve more complex and diverse patterns, such as landscapes and buildings in the background. Additionally, their approach only focuses on the face, so low-resolution processing is sufficient for their purpose, which does not apply to our work.

### 2.2. Object Detection and Segmentation

Many attempts have been made to leverage deep learning in computer vision for image classification, object detection, and object segmentation. Convolutional Neural Network (CNN)-based networks have been extensively researched to solve these problems, with AlexNet [16] being the starting point for many neural network structures. Subsequently, VGGNet [17] was widely used as a network, which increased the neural network’s depth compared to AlexNet but retained a simple structure. Moreover, R-CNN [18] was introduced based on AlexNet; R-CNN follows the basic structure of AlexNet but differs in that the input image has multiple object regions. Mask R-CNN [19] was developed for image segmentation, which locates the object’s position and then applies a mask to the precise area. An additional CNN checking whether each pixel belongs to the object was added to R-CNN. Models trained on datasets such as Celeb-A [20] and Places2 [21] using the Mask R-CNN structure are still commonly used for image segmentation. Recently, a number of works employ transformers for image segmentation, such as SegFormer [22], Mask2former [23], Mask DINO [24], OneFormer [25], MedSegDiff [26], and CMFormer [27].

This work utilizes prior image segmentation methods for people detection in acquired frames. However, using only these methods has some limitations when it comes to accurately detecting all regions of a person’s body. Certain areas of the person’s body, such as arms and feet, and some accessories, are occasionally missed in the predicted regions from them, which can negatively impact the results. Thus, novel solutions based on such methods are proposed to address these limitations and improve the accuracy of person detection in images.

### 2.3. Image Inpainting

Image inpainting is the process of restoring specific or partial areas within images using traditional or deep learning techniques. Traditional methods, such as the Alexandru–Telea [28] and Navier–Stokes [29] algorithms, typically rely on nearby pixel information to restore the missing parts. However, with the advancement of deep learning techniques, image inpainting using Generative Adversarial Networks (GANs) [30] has become popular, including Jiahui et al. [31], Edge-connect [32], and CM-GAN [33]. Recently, several studies have used transformers for image inpainting, such as MAT [34], TransInpaint [35], CMT [36], SyFormer [37], and WaveFormer [38]. Although such image inpainting methods work well for relatively simple patterns, they are limited when restoring complex areas, large holes, or those located on the sides of the image [7,8]. Additionally, the methods often require significant computing resources [8,9], making high-resolution processing difficult. While an image inpainting technique is also employed in this work, a method that minimizes the restored areas is newly proposed to improve the quality of the resulting images.

### 2.4. Key Differences from Prior Works

Table 1 compares our method and prior works. First, the proposed method differs from others in that it utilizes a few-second video as input to generate a human-removed image. Second, while it employs existing semantic segmentation methods to automatically detect specific objects (i.e., people in a picture), it devises a feature to fine-tune the detected object’s boundaries. Specifically, the proposed method exhaustively identifies human areas for removal through an elaborated dilation operation. Third, the identified regions of the objects to be removed undergo a region-minimizing process, which is also a significant difference from prior methods. Leveraging a few-second video as input allows us to minimize the regions that require recovery. It is highly likely to obtain natural image pixel information in the human-removed areas with complex background patterns from consecutive frames from the video, which is impossible with a single input image. Finally, the minimized regions are passed to the image inpainting step. Accordingly, the quality of images generated by our processing pipeline can be improved. Additionally, it has the potential to prevent the excessive consumption of computational resources.

## 3. System Overview

### 3.1. Design Considerations

This paper proposes Thanos, a system that generates images with only the background scenery or landmarks by removing people from photos taken in crowded areas. Two major design considerations for developing Thanos are presented below.

- High-quality image generation: The system should be able to generate satisfactory images for users. To achieve this, it should be able to accurately segment and crop human-related areas and then naturally restore the cropped areas. Additionally, the system should support resolutions of Full High Definition (FHD) or higher.
- Low user burden: The system should be able to produce desired results without imposing any additional burdens on users, such as manually selecting areas for removal or being involved in the image restoration process. It should automatically identify areas for removal and process images to achieve this.

### 3.2. The Proposed Approach

One straightforward approach to removing people from an image could be to segment and crop the area occupied by the people and then restore the cropped area using image inpainting methods. However, it was found that the approach had some issues. First, certain parts of people, such as accessories like shoes and bags or the edges of the person’s hands and feet, were not accurately segmented out. Second, image-restoring methods usually rely on the surrounding pixels, so the recovery quality is affected depending on such surrounding pixel information. For example, if the pattern around the cropped area is complex or the area is located at the edge of the image where surrounding information is scarce, the quality of the resulting image could be degraded. In addition, the state-of-the-art restoring algorithms based on deep learning may require high computational costs.

We present a multi-frame-based recovery region minimization approach to address the problems. Its key idea is to aggregate information partially available from multiple image frames to minimize the area to be restored. Our approach has two main advantages.

- By maximizing the utilization of actual pixel data from image frames, it is possible to minimize the use of restored image pixels. This improves the quality of the resulting images.
- By reducing the need for high-cost recovery operations, it is possible to decrease the computational resources required even though it should handle multiple frames.

To apply the proposed approach, users should take a short video of about 5 s at the location where they want to obtain a background image without people. Although taking a 5-s video might seem more burdensome than taking a picture, framing a shot to capture a great scene often takes several seconds or more, even when taking a single photo. Thus, it might be likely that taking a short video does not place an additional burden on users.

### 3.3. System Architecture

**Figure 1:**
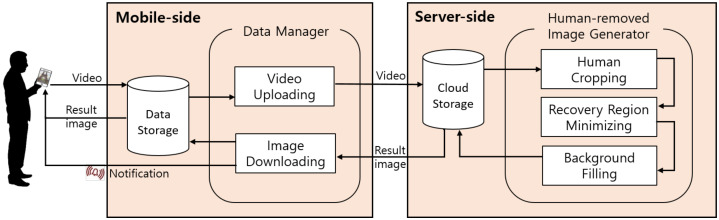
Overall architecture of the proposed system.

The architecture of the proposed Thanos system is shown in Figure 1. It consists of two major components: a mobile side and a server side.

Mobile-side: The mobile-side component is mainly responsible for interacting with a user. A short video of about 5 s is acquired from the user and stored in the system’s internal storage. When the user requests to process human removal, the Data Manager sends the videos to the server side. After human removal is completed on the server side, the Data Manager receives a resulting image. After the image downloading is complete, the Data Manager stores the image in the storage and notifies the user that the processing is complete so that he/she can view the result.

Server-side: The server-side component is responsible for generating human-removed images. The server-side component receives a video from the mobile side and extracts image frames for the video. It then stores one frame per second in the server’s storage. The Human-removed Image Generator in the server-side component recognizes when data to process are stored in the storage and begins processing once they are ready. It performs three main operations: human cropping, recovery region minimizing, and background filling. The resulting human-removed images are stored in the storage and then sent back to the mobile side.

### 3.4. System Scope and Limitations

In this paper, several assumptions are made to develop the proposed system. Firstly, it is assumed that the desired background scenery can be somewhat seen in the image frames captured from a few seconds of video. To illustrate this point, consider being at a famous tourist spot like the Eiffel Tower in France. People often wait a few seconds with their camera until fewer people are in the frame to take a clear picture of the Eiffel Tower.

Secondly, generating images without any people in the scene is our objective. However, there could be other situations besides removing people when taking photos. For example, someone may want to capture a picture of their family or friends with a landmark in the background. In this case, face detection and recognition can be used to keep specific people in the frame. Further study on these cases is left for future work.

Thirdly, cloud offloading is utilized to realize the system. It might be possible to perform all the processing on the user’s mobile device. However, generating high-quality images requires a high-accuracy human segmentation and recovery algorithm with complex deep learning networks, which consumes heavy computational resources. Additionally, processing high-resolution pictures on mobile devices can be a significant burden. These factors could result in longer processing times and negatively affect the user experience. Therefore, in this work, a cloud server is used to handle complex processing and high-cost computations that are challenging to perform smoothly on mobile devices.

## 4. System Design and Implementation

### 4.1. Processing Pipeline Overview

**Figure 2:**
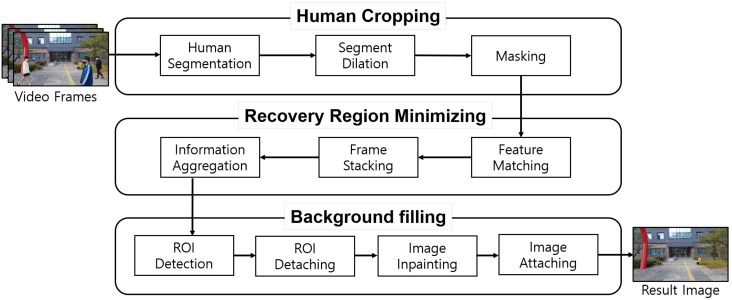
Image processing pipeline.

This section presents a novel processing pipeline to generate high-quality human-removed images. Figure 2 shows the processing pipeline developed for the Human-removed Image Generator shown in Figure 1. The pipeline consists of three modules: the Human Cropping module, the Recovery Region Minimizing module, and the Background Filling module.

The Human Cropping module aims to crop all human regions in each input frame. For this purpose, human segmentation is performed to identify human regions in the video frames. Then, segment dilation is performed to expand the segmented regions. Next, masking is applied to crop out the dilated human regions. After this process, the resulting frames with cropped human areas are passed to the Recovery Region Minimizing module.

The Recovery Region Minimizing module aggregates the information of the frames in which the human area is cropped. The operation aims to minimize the recovery region, achieving higher-quality human-removed images. To achieve this, it compensates for perspective differences between frames caused by slight movements of the camera. Then, it stacks the frames and, finally, aggregates the pixel information of each frame.

The Background Filling module is responsible for restoring the remaining empty regions in the output image from the Recovery Region Minimizing module. Firstly, ROI detection is performed to automatically identify the ROIs that require restoration, followed by ROI detaching to separate them. Next, image inpainting is applied to restore the detached ROI. Finally, image attaching is performed to place the restored ROIs back into their original positions, resulting in a final human-removed image.

Algorithm 1 describes the overall algorithm of human removal image processing. It sequentially employs image segmentation on each frame of the input few-second video to identify areas corresponding to humans. The resolution of each frame input for image segmentation is 1024 × 1024, and every fifth frame is utilized (Lines 1–3). Dilation operations are then applied to each human area, followed by the masking of these portions. The dilation ratio is set to 0.002 based on our experimental results (see Section 4.2) (Line 4–6). Subsequently, sequential feature matching is executed for the masked frames to ensure temporal alignment, and then frame stacking is performed accordingly (Lines 10–14). Information aggregation is then applied to the stacked frames to generate a single frame (Line 15). Finally, all ROIs that are still empty areas are detected from this single frame (Line 16). These ROIs are detached, and inpainting is performed for each of them individually. These ROIs are detached, and inpainting is performed for each of them individually. Each ROI is detached at 512 × 512 resolution, and image inpainting is carried out after resizing them to 256 × 256 (see Section 4.4). Then, they are restored to their original positions, generating an image without humans (Lines 17–21).
**Algorithm 1:** Human removal image processing
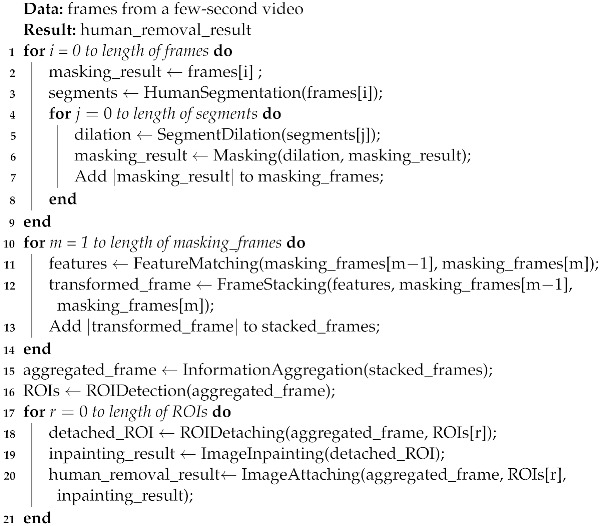


We discuss the algorithm’s complexity in terms of the number of operations involved in processing input image frames from a few-second video. First, the number of human segmentation operations equals the number of frames predefined as a constant. Segment dilation and masking operations are proportional to the number of segmented areas, which increases depending on the number of people in an image frame. Second, the number of feature-matching and frame-stacking operations also equals the number of frames. Third, ROI detaching and image inpainting operations are proportional to the number of detected ROIs. The worst-case scenario would be when the number of people is high and there is no movement of people in the input video. More people in a frame increases the number of segmented areas, resulting in a higher cost of segment dilation and masking operations. If people are stationary, there would not be much change in the segmented areas for the people across consecutive frames. Thus, even after frame stacking, the regions requiring background filling would likely match the segment areas identified as people in the image segmentation process. In this case, the number of ROIs increases, leading to an increase in image inpainting operations.

In this paper, Mask R-CNN [19] and EdgeConnect [32] are employed for human segmentation within the Human Cropping module and image inpainting in the Background Filling module, respectively. This choice is based on the performance evaluation of combinations of several widely used models (see Section 5.2). Note that the proposed processing pipeline is not bound to specific techniques and is designed to adopt new human segmentation and image inpainting models.

### 4.2. Human Cropping

**Figure 3:**
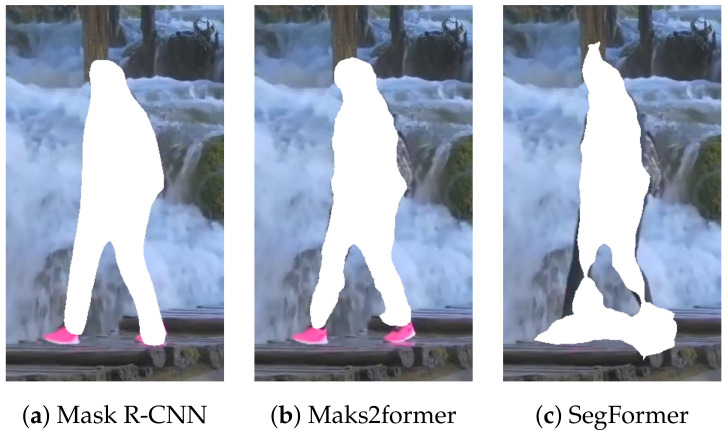
Human cropping examples using image segmentation methods without dilation (Images were captured on Touropia (https://www.youtube.com/@touropia), accessed on 14 April 2024).

**Figure 4:**
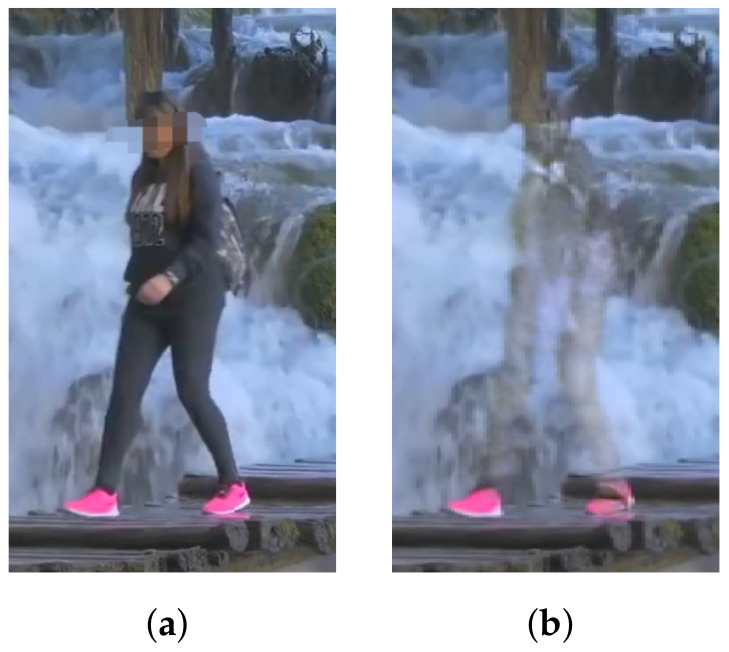
Human removal without dilation (Images were captured on Touropia (https://www.youtube.com/@touropia), accessed on 14 April 2024). (**a**) Before human removal. (**b**) After Human removal.

It is necessary to determine where humans are located within an image to remove humans from the image. A straightforward approach is to use image segmentation methods. However, simply applying them could result in imprecise identification of the human regions, negatively impacting the quality of the resulting image. Figure 3 shows human cropping examples using several widely used image segmentation techniques such as Mask R-CNN [19], Mask2former [23], SegFormer [22]. It can be seen that some parts of the humans, such as legs and shoes, are not accurately segmented. These parts remain even after the humans are removed and the segmented regions are restored (see Figure 4). Users are highly likely to consider such a result quite unnatural, which degrades the user’s satisfaction.

**Figure 5:**
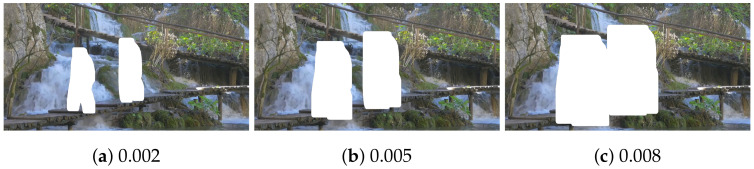
An example of segmentation results with different dilation ratios (Images were captured on Touropia (https://www.youtube.com/@touropia), accessed on 14 April 2024).

Expanding human regions predicted by a segmentation model is necessary to address the above-mentioned issue. A simple way is to dilate the regions by a fixed number of surrounding pixels. However, this may cause a problem. For example, enlarging relatively smaller human areas can be excessive if the number of pixels is set to cover large human regions sufficiently. Thus, a policy to determine the degree of dilation of human areas proportionally to their size is adopted. The larger the segmented human regions are, the more surrounding pixels are dilated. Here, choosing an appropriate dilation ratio is needed to balance the accuracy of clearly segmenting out human regions and the size of the areas to be recovered. If the ratio is too large, too much dilation is performed far beyond human parts, thereby making areas to be restored much larger. On the other hand, a too small ratio results in insufficient dilation, causing human regions not to be segmented thoroughly. Figure 5 shows an example of segmentation results depending on the ratio.

**Figure 6:**
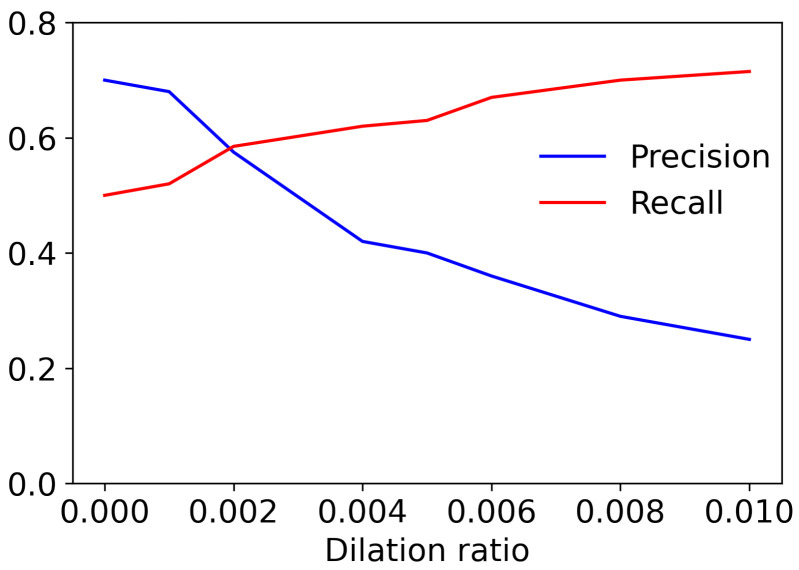
Precision and recall with dilation ratios.

In this work, an appropriate dilation ratio is determined empirically. It is examined how different ratios affect final segmentation results for this purpose. The pre-trained Mask R-CNN model is used for the initial segmentation before dilation. Figure 6 shows the average recall and precision with different dilation ratios. As the ratio increases, false negatives, i.e., the area that is not segmented even though it belongs to human regions, decrease, thereby increasing the recall. However, false positives also increase, which may negatively impact the image quality. Increasing false positives decreases the precision. For balanced performance, the dilation ratio is set to 0.002.

### 4.3. Recovery Region Minimizing

**Figure 7:**
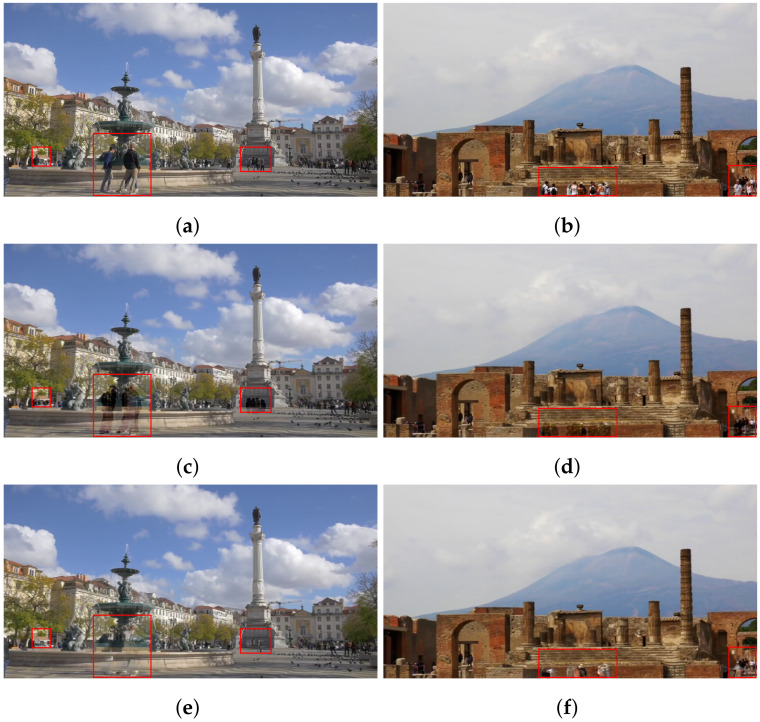
Original images (**a**,**b**), and image inpainting results by EdgeConnect (**c**,**d**) and MAT (**e**,**f**) (Images were captured on Touropia (https://www.youtube.com/@touropia), accessed on 14 April 2024).

The masked areas should be filled in as the next step of human cropping. The straightforward approach is to recover these areas using image inpainting techniques. However, simply employing these techniques poses some problems. Figure 7 shows the results of removed human area restoration with image inpainting techniques such as EdgeConnect [32] and MAT [34]. In comparison to the original images (Figure 7a,b, we can see unnatural areas where there are people as shown in the red boxes in Figure 7c–f. The problem could occur when the areas to be restored have complex patterns or are positioned at the edges of the scene, where there may be limited reference information available.

To resolve the problem, a method that utilizes multiple image frames from a short video is employed to generate human-removed images. Through this, the empty areas to be restored with an image inpainting model can be minimized. It is advantageous because information obscured by a person in a frame can be obtained from some subsequent frames where the person moves. For this purpose, the consecutive frames containing masked human regions are stacked sequentially, and the information of every pixel in the frames is aggregated.

**Figure 8:**
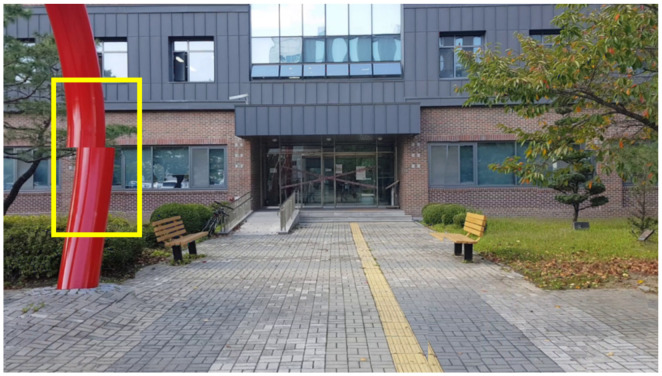
Frame stacking result without feature matching.

However, simple frame stacking might lead to undesirable images due to the mismatch in perspective among consecutive frames. When taking a video with a hand-held smartphone, slight shaking and movement of the hand can cause the perspective of the frames in the video to vary. If these variations are not considered during the frame stacking process, they result in misalignment, as shown in the yellow box in Figure 8.

To handle the issue, frame-to-frame feature matching is performed. Generally, consecutive frames from a short video are likely to be quite similar, especially when shooting a video at a specific landmark for a short time (e.g., 5 s). It is highly probable that the same feature points exist in these frames. Using a feature-matching algorithm, the perspective of the frames is transformed to align them, and the aligned frames are stacked. Note that several feature matching algorithms are considered, such as Orientated FAST and Robust BRIEF (ORB) [39], Speeded Up Robust Feature (SURF) [40], and Scale-Invariant Feature Transform (SIFT) [41]. Among them, ORB is used in our processing pipeline because it shows a relatively short processing time compared to other algorithms with similar performance.

### 4.4. Background Filling

**Figure 9:**
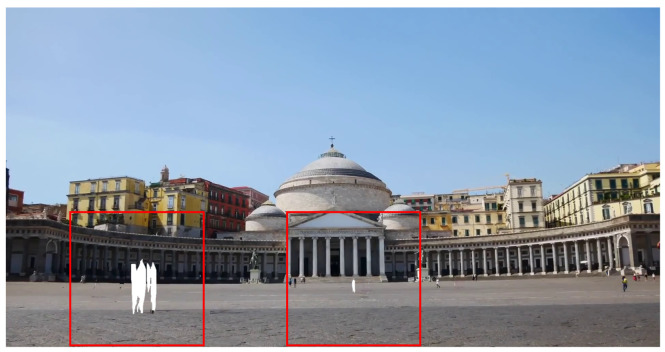
Example of remaining empty area after frame stacking (Images were captured on Touropia (https://www.youtube.com/@touropia), accessed on 14 April 2024).

The regions that are still empty, even after the recovery regions are minimized through frame stacking, should be restored. In cases where there is little or no movement of people within a few seconds of the video, empty areas may remain in the image, as shown in the red rectangle in Figure 9. Generally, GAN-based image inpainting techniques could be utilized, which have shown good performance [32]. However, substantial resources are required to train GAN-based image restoration models and generate images using them. Thus, they are usually trained at 256 × 256 resolution; the pre-trained models are provided at such resolution. However, the typical resolution of smartphone pictures is much higher.

**Figure 10:**
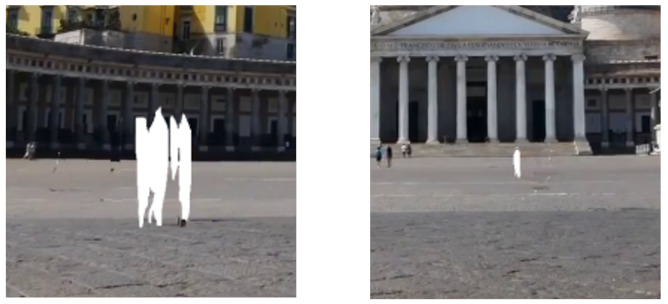
Detached images for image inpainting (Images were captured on Touropia (https://www.youtube.com/@touropia), accessed on 14 April 2024).

To resolve this issue, only a small portion of the image is detached and restored, including empty areas that require recovery, rather than using the entire image for restoration. Specifically, all ROIs for inpainting are detected, as shown in Figure 9. The detected ROIs are detached, as shown in Figure 10. Then, the detached images are input into an image inpainting model to restore them.

The ROIs at 512 × 512 are detached, although the model’s input size is 256 × 256. It is because using an ROI of 256 × 256 size, which is only about 1/32 of the FHD size, can cause a problem in some cases. For example, the people in the image may be larger, sometimes even larger than the ROI size. In this case, a restored image may appear unnatural due to limited information surrounding empty areas. Thus, a larger ROI, i.e., 512 × 512, about 1/8 of the FHD size, is used. Our collected data hardly found cases where a person in the image occupied more than 1/8 of the entire picture.

The final processing step is performed with the detached images, i.e., inpainting and attaching. Before inpainting, the images are resized to a 256 × 256 size, suitable for an image inpainting model’s input. The restored images from the model are resized back to a 512 × 512 size. They are then attached to their original positions in the entire picture. As a result, natural-looking photos with no people in them are obtained.

## 5. Evaluation

This section presents the evaluation of Thanos in two aspects. First, the proposed processing pipeline is evaluated in terms of image quality and processing latency. Second, Thanos is compared with well-known existing applications, Retouch-photos, and Samsung object eraser.

### 5.1. Experimental Setup

**Figure 11:**
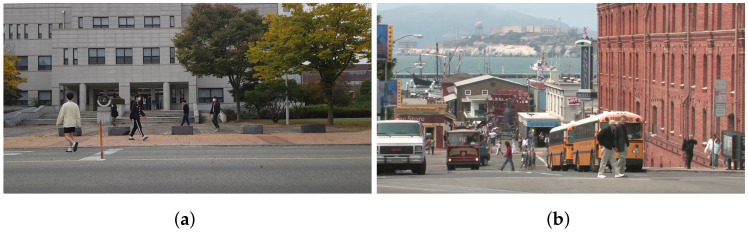
Example of original image frames including humans. (**a**) Example image captured by researchers. (**b**) Example image downloaded from Touropia.

For evaluation, 20 videos were collected, which were 3–5 s long; some were recorded by researchers (e.g., Figure 11a) and the others were downloaded from Touropia Youtube channel (https://www.youtube.com/@touropia, accessed on 14 April 2024) (e.g., Figure 11b). We specifically target the case when an input video contains a moderate number of people showing natural movement in a real-world scenario. We carefully considered several factors in preparing the input data workload to ensure the validity and generalizability of the experimental results. Firstly, the videos recorded by researchers were captured in authentic outdoor settings on a university campus, reflecting people’s natural behavior. It ensures that our data accurately represents real-world conditions and behaviors. Secondly, to expand the scope of our evaluation, we carefully selected videos from YouTube featuring people moving naturally in diverse famous locations. Specifically, we selected videos where people were moderately active, as videos with no people moving would not significantly differ from simple image inpainting. Additionally, we excluded videos with excessive crowds obstructing landmark visibility. The videos were divided into two cases to measure the performance with the proportion of the human part in a frame. Based on our observation of the videos, a threshold for the ratio of human regions is set to 2%. If it is less than the threshold, we consider it the small case and the others (more than 2%) the large one.

As a metric for image quality, we use the FID score, which is widely used to assess the quality of images created by generative models. To examine the FID of human-removed images, about 30,000 images from the places2 dataset [21] were used. Note that the FID score represents the statistical distance between the actual image dataset and the generated image. The lower the number, the more similar it is to the original data sample.

### 5.2. Thanos Performance

#### 5.2.1. Image Quality

We evaluate the quality of generated images in three aspects, i.e., the effect of the proposed operations, the effect of dilation ratios, and the effect of the proportion of human regions. First, the image quality is assessed by examining the effect of the presented operations, i.e., segment dilation and frame stacking, to minimize the recovery region. To quantify the impact of each process, we implemented three variants of our method, Single frame without dilation, Single frame with dilation, and Thanos, in which we applied each technique in turn, and we compare their performance. Specifically, Single frame without dilation is a baseline method that does not apply the proposed approach, i.e., segment dilation and frame stacking. Note that it represents a method using only existing segmentation and image inpainting models in previous work. Single frame with dilation is the method that applies segment dilation only without frame stacking. Finally, Thanos is the proposed method. The first two methods use the first frame of a video as an input for image segmentation and restoration.

**Figure 12:**
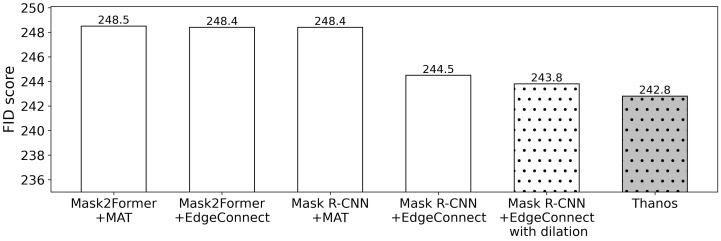
Performance breakdown.

White color bars in Figure 12 present the FID score measurements for several baseline methods (i.e., Single frame without dilation) that merely utilize combinations of image segmentation models such as Mask R-CNN [19] or Mask2Former [23] and image inpainting models such as EdgeConnect [32] or MAT [34], including both CNN and GAN-based earlier models and transformer-based recent models. Note, that we considered these models as candidates for integration into Thanos to assess our proposed pipeline performance. Our objective was to investigate how various models with their respective strengths and weaknesses would impact the performance within our pipeline. The result demonstrates that the combination of Mask R-CNN and EdgeConnect shows better image quality than other alternatives. Thus, Thanos’ processing pipeline adopts them and applies the proposed techniques, i.e., segment dilation and frame stacking, to minimize the recovery region.

Figure 12 also shows that Thanos provides better quality than the other variants. The FID scores for the best-performing combination of image segmentation and inpainting models, Mask R-CNN+EdgeConnect, the same combination with added dilation, and finally, Thanos with all proposed techniques applied are 244.5, 243.8, and 242.8, respectively. Note that these FID scores are the average numbers of the FID values obtained with the 20 video data mentioned above. It can be seen that the quality of the resulting images improves as the operations are applied. The segment dilation operation helps remove human areas such as shoes and fingertips more thoroughly, and the frame stacking process reduces the region to restore. As a result, Thanos improves image quality.

**Figure 13:**
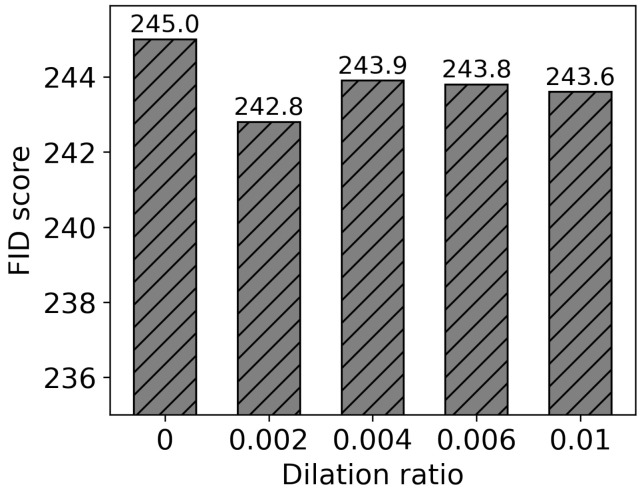
FID score with dilation ratios.

Figure 13 shows the FID scores depending on the dilation ratio. The FID score is the smallest when it is 0.002. It increases again and then gets saturated as the ratio gets greater. Generally, performance is better when segment dilation is applied than when it is not. In some cases, human parts such as fingertips and shoes remained even after human segmentation without segment dilation. These parts degrade image quality. As mentioned above, if the dilation ratio is too small, the human area is not adequately segmented, resulting in decreased image quality. On the contrary, if it is too large, the quality decreases because more segmented regions need to be recovered. Setting an appropriate ratio (0.002 in this paper) is essential for the quality of generated images.

Table 2 shows FID scores with the applied operations and the proportion of human regions. In all cases, Thanos generates better-quality images. In detail, the FID score is lower when the ratio of human areas is relatively small than when it is significant. This is mainly because the lower the human-region ratio, the smaller the area needs to be restored. However, Thanos still achieves a lower FID score than the other alternatives.

#### 5.2.2. Processing Latency

We assess the processing latency to examine Thanos’ computational overhead. The baseline method that does not apply segment dilation and frame stacking (i.e., single frame without dilation introduced above) is used for comparison. As mentioned above, the first frame of a video is used as input. Table 3 shows the hardware setup used for the experiment.

Table 4 shows the average processing latency. The latency of Thanos is comparable with the method using only a single frame, although Thanos processes multiple frames to generate an image. If multiple frames are used, the latency of the segmentation operation increases in proportion to the number of frames. However, the recovery region is minimized through frame stacking so that the cost of the inpainting operation is effectively reduced. As a result, Thanos generates better-quality images without increasing the processing latency.

Interestingly, the average processing latency is larger when the ratio of human areas is small than when it is large. In the small case, people in a frame were far from the camera and small in the background. It will likely result in more ROIs to process, which involves more ROI detaching, inpainting, and attaching operations.

### 5.3. Comparison with Existing Applications

Thanos is compared with two existing applications, i.e., Samsung object eraser and Retouch-photos, which are well-known applications for removing unwanted objects in taken pictures. Both apps require users to select areas to remove, and they restore the chosen regions. Accordingly, the human parts were manually set using the first frame of the video to obtain human-removed images with these apps. The image quality is assessed using the FID scores of the images by Thanos, Samsung object eraser, and Retouch-photos.

**Figure 14:**
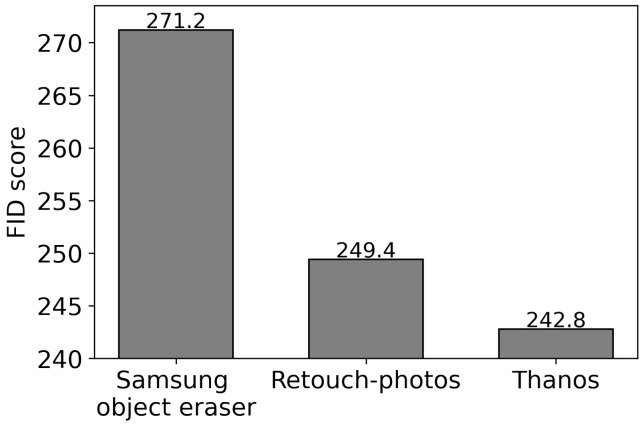
FID score comparison.

**Figure 15:**
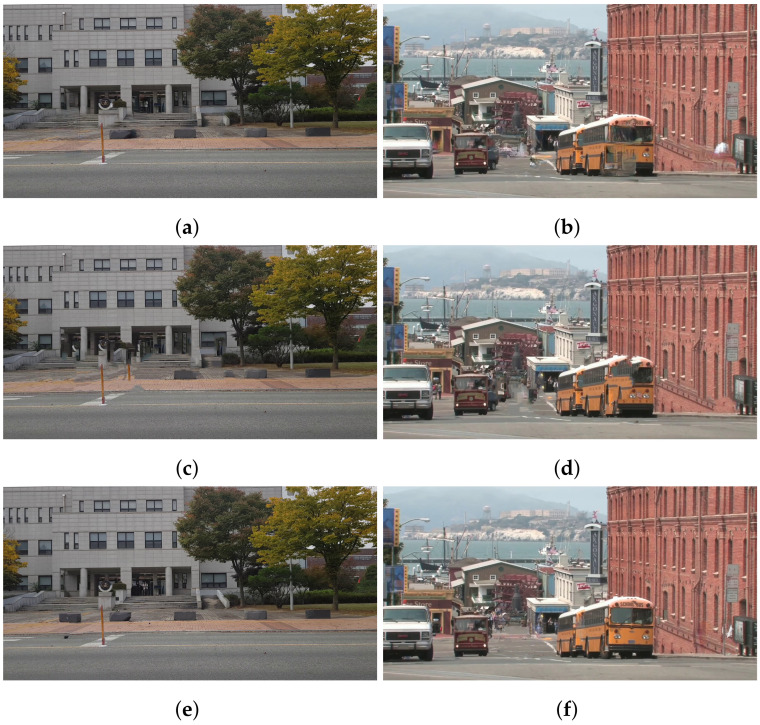
Example of results by Samsung object eraser (**a**,**b**), Retouch-photos (**c**,**d**), and results by Thanos (**e**,**f**).

Figure 14 shows the FID score of Thanos is lower than alternatives. It means Thanos generates more natural human-removed images than Samsung object eraser and Retouch-photos. Such apps restore the user-specified areas in a single image. In some cases, the quality of restored regions may not be good enough. For example, if the area to be recovered involves a complex pattern, such as a building in Figure 11a and a yellow bus in Figure 11b, it is not restored correctly, as shown in Figure 15a–d. On the other hand, Thanos utilizes multiple frames, and thus, it is likely to acquire information about some occluded areas in a frame from the subsequent frames. Accordingly, Thanos reduces the size to restore and generates better-quality images, as shown in Figure 15e,f.

The effect of the proportion of human regions is further examined. Table 5 shows Thanos achieves lower FID scores than Samsung object eraser and Retouch-photos regardless of the ratio of human areas. As discussed earlier, the FID score is smaller when the human region ratio is small than when it is large.

## 6. Limitation and Future Work

There are still several limitations that need to be addressed in future work. First, this study focused on removing people from the scene of captured images. However, users may have various needs. For example, some users may want to keep themselves or specific people in the image. This could be achieved by identifying the particular people and not cropping them. Specifically, face recognition [42] could be utilized to segments identified as people by semantic segmentation to automatically determine if each segment corresponds to individuals users want to keep in the image. The addition of features reflecting user needs is left for future work.

Second, the proposed system relies on external resources. This could lead to an overall increase in processing latency and may raise privacy concerns. Addressing this issue could be achieved to enable end-to-end processing on mobile resources. It could involve light-weighting computationally intensive techniques, such as image inpainting [43,44]. Additionally, adopting pipeline optimization techniques could be essential. For instance, caching [45] can be employed in scenarios where there is minimal frame-to-frame variation within a few seconds of video to prevent redundant computations. However, it is essential to carefully consider a balance between the quality of the generated images and resource consumption.

Third, some features of the proposed system were implemented using pre-trained models from previous studies. To enhance the performance of Thanos, collecting new data for training or fine-tuning models that consider our specific context may be necessary. Designing a new training architecture could also be considered as future work.

Fourth, we need to consider the broader implications of technology that automatically removes humans from images. It includes understanding the technical aspects, ethical considerations, and potential for misuse. For instance, there may be concerns about the credibility and truthfulness of media due to image manipulation and the creation of opportunities for disseminating false information. We should take comprehensive measures to minimize any potential side effects that might arise from the proposed technology. We consider this to be an important aspect of future work.

## 7. Conclusions

In this paper, we present Thanos, a novel system to generate a human-removed image using a short video clip in crowded places. To this end, a novel processing pipeline is devised to make human removal automatic, accurate, and efficient. Several experiments are presented to evaluate the performance of Thanos in terms of image quality and processing latency. Thanos is also compared with an existing application.

## Figures and Tables

**Table 1:** Comparison with related works.

	Input	Output	Automatic Target Object Detection	Fine-Tuning Target Object Area	Recovery Region Minimizing
Mask R-CNN [19]	Image	Semantic segmented image	Yes	No	N/A
SegFormer [22]	Image	Semantic segmented image	Yes	No	N/A
Mask2former [23]	Image	Semantic segmented image	Yes	No	N/A
Mask DINO [24]	Image	Semantic segmented image	Yes	No	N/A
OneFormer [25]	Image	Semantic segmented image	Yes	No	N/A
MedSegDiff [26]	Medical image	Semantic segmented image	Yes	No	N/A
CMFormer [27]	Image	Semantic segmented image	Yes	No	N/A
Jiahui et al. [31]	Damaged image	Recovered image	No	No	No
Edge-connect [32]	Damaged image	Recovered image	No	No	No
CM-GAN [33]	Damaged image	Recovered image	No	No	No
MAT [34]	Damaged image	Recovered image	No	No	No
TransInpaint [35]	Damaged image	Recovered image	No	No	No
CMT [36]	Damaged image	Recovered image	No	No	No
SyFormer [37]	Damaged image	Recovered image	No	No	No
WaveFormer [38]	Damaged video	Recovered video	No	No	No
Shetty et al. [11]	Image, text	Specific object-removed image	Yes	Yes	No
Dhamo et al. [12]	Image, text	Semantic manipulated image	Yes	No	No
Din et al. [13]	Image	Mask-removed face image	Yes	Yes	No
HiMFR [14]	Image	Mask-removed face image	Yes	No	No
Sola et al. [15]	Image, text	Mask-removed face image	Yes	No	No
Thanos (ours)	A few-second video	Human-removed image	Yes	Yes	Yes

**Table 2:** FID score with the applied operations and the proportion of human regions.

	Single Frame without Dilation	Single Frame with Dilation	Thanos
Small	257.0	257.4	254.7
Large	306.7	309.2	299.8

**Table 3:** Experimental hardware setup.

CPU	Intel i7-7700
GPU	NVIDIA Geforce RTX 2080Ti
RAM	32 GB
Compiler	PyTorch 1.13.1 (with CUDA(10.2) + cuDNN)

**Table 4:** Average processing latency.

	Single Frame without Dilation (ms)	Thanos (ms)
Small	761	754
Large	644	663

**Table 5:** FID score with the proportion of human regions.

	Samsung Object Eraser	Retouch-Photos	Thanos
Small	301.8	260.1	254.7
Large	311.1	306.6	299.8

## Data Availability

The raw data supporting the conclusions of this article will be made available by the authors on request.
